# Bacterial Translocation as a Source of Postoperative Fever After Revision of a Previous Duodenal Switch for Super-morbid Obesity: A Case Report

**DOI:** 10.7759/cureus.37722

**Published:** 2023-04-17

**Authors:** Panagiotis Sakarellos, Markos Despotidis, Adam Mylonakis, Spyridon Davakis, Ilias Vagios, Eleandros Kyros, Andreas Alexandrou

**Affiliations:** 1 First Department of Surgery, Laikon General Hospital, National and Kapodistrian University of Athens, Athens, GRC

**Keywords:** postoperative fever, bariatric surgery, revision surgery, duodenal switch, bacterial translocation

## Abstract

Bacterial translocation is defined as the invasion of gut bacteria or bacterial products to the systemic circulation via permeation through the gastrointestinal mucosal wall. In this article, we present the case of a patient with postoperative fever of unknown origin which was attributed to bacterial translocation after revisional surgery due to malabsorptive complications after an initial duodenal switch for super-morbid obesity.

## Introduction

Bacterial translocation is defined as the invasion of gut bacteria or bacterial products through the gastrointestinal (GI) wall, leading them into normally sterile tissues and possibly causing a septic clinical syndrome. This can occur through various mechanisms, including bacterial overgrowth, host immunosuppressive diseases, and the dysfunction of the gut epithelium [1]. Intestinal cells have anti-invasive mechanisms which prevent the penetration of microbes through the mucosal wall. These include the tight junctions (TJs) between cells [2], which prohibit the passage of microbes between them and the secretion of mucins. In this way, a thick extracellular glycoprotein wall is produced, which has multiple roles in preventing contact between GI cells and microbes but also traps the secreted IgA proteins and naturally produced antibiotics (called antimicrobial peptides, AMPs), which help the recognition of microbes and the deactivation of numerous toxins [3,4]. Despite a plethora of protective mechanisms, various conditions can alter the balance between the microbes and these protective mechanisms, making the GI wall permeable to microbes. As a result, they migrate within the satellite lymph nodes [5], through them into the blood circulation, and, finally, into various organs leading to systemic inflammatory responses [6]. Here, we present a case of fever due to bacterial translocation in what can be a not infrequent clinical scenario in morbidly obese patients who have undergone malabsorptive operations and have to be reoperated due to malnutritional complications.

## Case presentation

**Figure 1:**
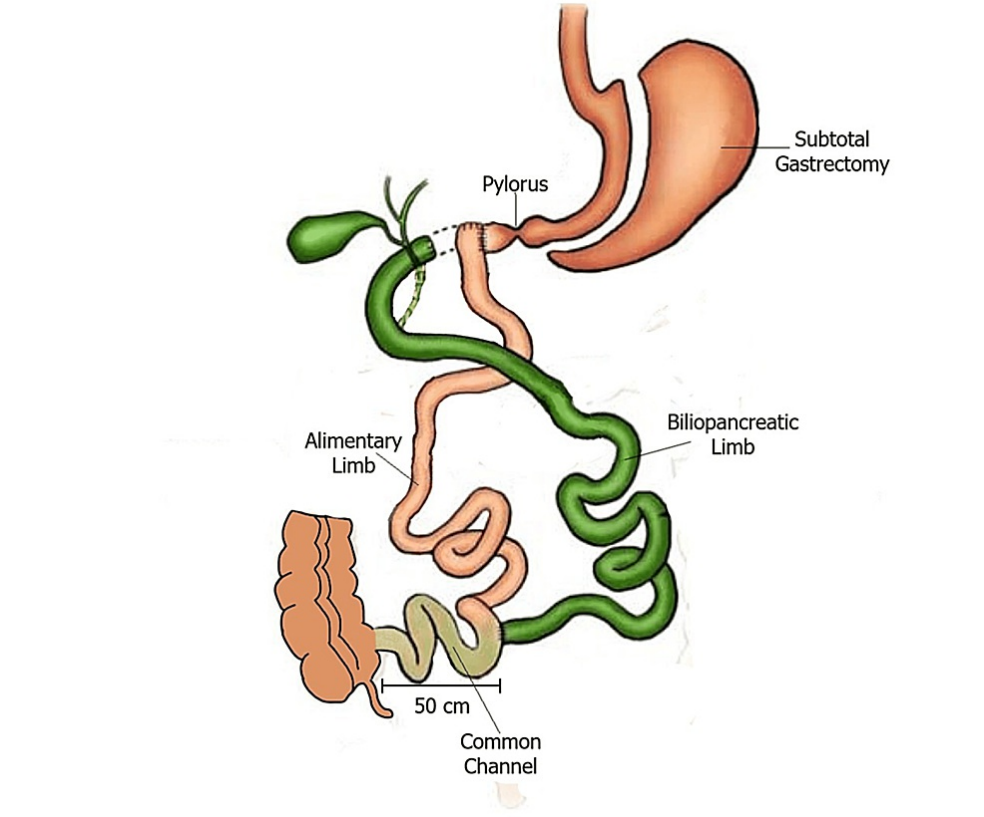
The duodenal switch procedure. Based on a figure published by Anthone et al. [7].

A 50-year-old male patient with a surgical history of duodenal switch three years ago (Figure 1) was admitted to our department due to nutritional complications. On physical examination, he was profoundly malnourished. The patient had a height of 1.78 m and a body mass index (BMI) of 22.7 kg/m^2 ^at the time of admission. He also reported nausea and vomiting after meals. Laboratory tests revealed hypocalcemia (serrum Ca^2+^ = 7.6 mg/dL, normal range = 8.6-10.2 mg/dL), hypoalbuminemia (serrum albumin = 29 g/L, normal range = 35-50 g/L), anemia (hematocrit = 32.6%, normal range = 40-54%), and low levels of ferrum (serrum Fe^2+^ = 29 g/L, normal range = 35-50 g/L), vitamin B12 (serrum B12 = 180 pg/mL, normal range = 223-925 pg/mL), and ferritin (serrum ferritin = 8 ng/mL, normal range = 30-400 ng/mL). Repeated upper GI endoscopies revealed an almost complete obstruction of the duodenoileal anastomosis.

**Figure 2:**
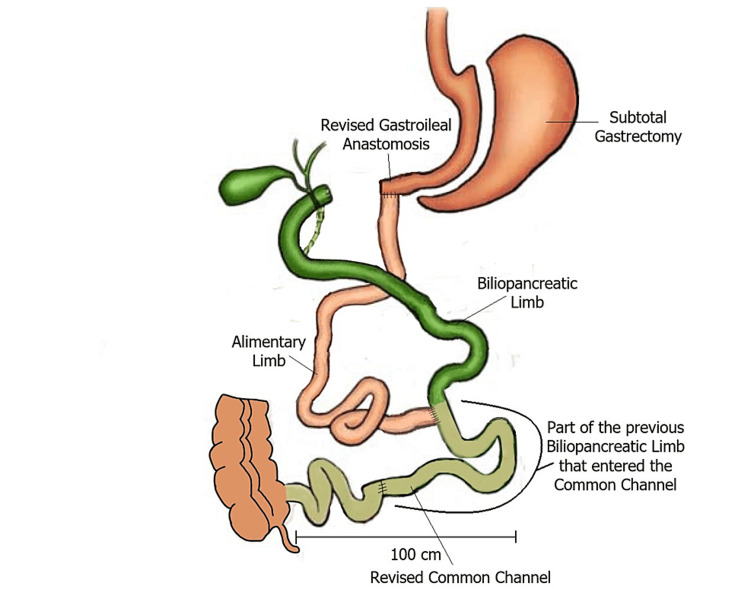
The revised duodenal switch procedure. Based on a figure published by Anthone et al. [7].

The patient underwent an exploratory laparotomy. The duodenoileal anastomosis was taken down and a new one was constructed. The common loop was measured and found to be extremely short, approximately 50 cm. The decision was made to take down the jejunoileal anastomosis and reconstruct it in a manner that would elongate the common loop by 50 cm more. The alimentary limb was anastomosed more proximally to the biliopancreatic limb, thus elongating the common loop by adding part of the biliopancreatic limb into it (Figure 2).

The operation was completed uneventfully. Upon resuming oral feeding, the patient started suffering from fever and diarrhea. Fever was refractory to specific antibiotic treatment, guided by blood culture tests, which always revealed multi-sensitive enterobacteria. Despite thorough whole-body scanning, including gallium scanning and computed tomography (CT) of the abdomen and thorax, no origin was traced. At that time, we assumed that fever might be attributed to bacterial translocation due to the re-establishment of oral feeding in a jejunal loop that had been for quite some time excluded from food circulation. Oral rifampicin (300 mg three times a day) was started to minimize the intraluminal bacterial load. The patient became afebrile in the first 24 hours and soon diarrhea also improved. The patient was discharged after 72 hours and remained well. He received oral rifampicin for other 30 days, and a lactose-free diet was recommended to the patient as a precautionary measure to exacerbate GI symptoms. Six months postoperatively he gained 10 kg of body weight (new BMI = 25.9 kg/m^2^), and all metabolic indices were normalized.

## Discussion

Although a duodenal switch is not as common a bariatric procedure as sleeve gastrectomy or Roux-en-Y gastric bypass, it is a very useful procedure as far as very severe obesity is concerned [8]. In this report, we present a case of postoperative fever due to bacterial translocation after revisional surgery of a duodenal switch. In fact, cases of severe bypass enteritis have been reported after jejunoileal bypass [9]. The main difference between biliopancreatic diversion and jejunoileal bypass, a procedure with high morbidity and mortality used in the past for super-morbid obesity, is that there is no *blind *loop. However, dietary changes, loss of ordinary enteric peristalsis, and bacterial overgrowth can also occur after bariatric procedures such as a duodenal switch. In fact, functional gut shortening may be the main risk factor for intraluminal bacterial overgrowth [10]. Enteric obstruction, a *blind *loop, and functional gut shortening can all contribute to bacterial overgrowth [9,10]. In our case, the obstruction of the duodenoileal anastomosis led to irregular propulsion of food to the alimentary loop as well as to the common loop. Additionally, the alimentary loop was excluded from bile circulation, while the biliopancreatic loop was excluded from food circulation. The reoperation, consequently, led to functional and structural changes in the GI tract. In fact, part of the small intestine, in which bacterial overgrowth and dysfunction of immune mechanisms had probably already been established, was again reunited with the rest of the GI alimentary loop where bacterial overgrowth and dysfunction of immune mechanisms had probably already been established. As a result, the postoperative period was complicated by bacteremia with an ensuing fever that could be managed efficiently only with oral antibiotic treatment to minimize the bacterial load exactly at its source, the gut lumen. Probiotics have also been considered as a way of controlling bacterial overgrowth [11]; however, in our case, oral rifampicin alone led to an immediate improvement in the clinical state of the patient. The conclusion that this ongoing fever was attributed to gut bacterial overgrowth was excluded mainly because no other site of infection was found, while the patient immediately improved after the treatment with rifampicin. However, because during the revisional surgery, a new jejunoileal anastomosis was created without the removal of any part of the small intestine, no histological evidence of any pathologic changes could be obtained. A hydrogen breath test was considered but not performed due to the rapid improvement in the patient’s condition after the initiation of rifampicin.

Small intestinal bacterial overgrowth is a well-defined consequence of bariatric procedures. The diagnosis is mainly made either by cultures of small bowel fluid or by the measurement of exhaled hydrogen and methane following the ingestion of a sugar substrate such as glucose or lactulose [12]. It can present with various symptoms, sometimes even with severe sub-nutrition [12,13]. Its treatment is based on antibiotic therapy, while revisional surgery may also be needed [14,15]. In our case, the small intestinal bacterial overgrowth that had contributed to the malnutrition of the patient also resulted in postoperative fever after the revisional surgery. Because of high suspicion, empiric treatment with antibiotics was given resulting in immediate improvement of the patient. With this report, we aim to emphasize the clinical significance of probable changes in gut microbiota, as well as the chance of bacterial permeation into circulation through the compromised intestinal barrier due to chronic deactivation after bariatric procedures that differentiate normal GI tract continuity.

## Conclusions

Bariatric procedures affecting the continuity of the GI tract may often lead to gut microbiota imbalances as well as to changes in the mucosal permeability that can have a notable clinical significance leading to infectious complications. Parts of the intestine previously excluded from the alimentary loop which become part of it after revisional surgeries may also contribute to it. Clinicians should always consider these when dealing with episodes of fever, diarrhea, and abdominal pain in patients who underwent revision of malabsorptive procedures with the shortening of a long biliopancreatic limb due to nutritional deficiencies.
